# The welfare of farmed Nile tilapia: a review

**DOI:** 10.3389/fvets.2025.1567984

**Published:** 2025-05-06

**Authors:** Wasseem Emam, Helen Lambert, Culum Brown

**Affiliations:** ^1^Institute of Aquaculture, University of Stirling, Stirling, United Kingdom; ^2^Faculty of Veterinary Medicine, Complutense University of Madrid, Madrid, Spain; ^3^Ethical Seafood Research, Glasgow, United Kingdom; ^4^Animal Welfare Consultancy, Kingsteignton, United Kingdom; ^5^School of Natural Sciences, Macquarie University, Sydney, NSW, Australia

**Keywords:** *Oreochromis niloticus*, fish welfare, fish behavior, ethology, aquaculture

## Abstract

Nile tilapia are, by absolute number of individuals, the most farmed species of fish today, yet we know little about how common husbandry practices impact their welfare. Despite their global importance, there is a notable lack of detailed, species-specific welfare guidelines for tilapia farming. This gap reflects the scarcity of research-based recommendations on appropriate breeding conditions, environmental parameters, and handling methods that fully consider their biology and behavioural ecology. This review explores key dimensions of Nile tilapia biology and the implications of commercial aquaculture practices on their welfare. Topics covered include common grow-out housing systems, water quality, stocking density, environmental enrichment, feeding practices, handling, transportation, and slaughter. The paper underscores the importance of developing species-specific welfare parameters and management practices to meet the welfare needs of these animals. Specifically, it describes the most common grow-out housing systems and how parameters inherent to those systems, such as stocking density and environmental enrichment, impact Nile tilapia welfare. The review singles out capture and slaughter processes as particularly detrimental to tilapia welfare and offers insight into how evidence-based approaches can enhance welfare in commercial farming operations.

## 1 Introduction

There were an estimated 124 billion finfish farmed and slaughtered in 2019, a 9-fold increase from 1990 (1). At approximately 7–16 billion individuals farmed per year, Nile tilapia (*Oreochromis niloticus*) constitute the single most commonly farmed species of fish in the world (110). Yet, despite their numbers and recognised sentience, there are numerous widespread welfare issues in tilapia aquaculture such as high mortality and morbidity rates (2, 3). Tilapia are one of the most easily farmed groups of fish as they are physiologically hardy, adaptable in feeding, and spawn frequently (4). Of the over 22 different species of tilapia farmed globally, Nile tilapia are the most commonly farmed. This is due to their fast growth, large final sizes and broad environmental tolerance compared with other species of the genus (4, 5). As recently as 2018, Nile tilapia accounted for 75% of the global tilapia production (6). Although Nile tilapia originate in northern and eastern Africa, they are now commonly farmed throughout the African continent, South America, and across Asia. The top three producing countries are currently China, Indonesia and Egypt (7).

Despite the scale at which tilapia are farmed, to the authors' knowledge, there are no stand-alone public or private sector animal welfare standards for farmed Nile tilapia currently in existence other than a course run by the FAI Academy, which incorporates welfare indicators and practices for tilapia (8). Furthermore, welfare assessment protocols for tilapia have only recently been released [e.g., (9)]. Both the Aquaculture Stewardship Council (ASC) and the Global Aquaculture Alliance's (GAA) Best Aquaculture Practices (BAP) certification schemes have sections on animal welfare in their tilapia standard but both miss many key components. Moreover, Naylor et al. (10) estimated that only 11% of globally produced tilapia come from certified farms (i.e., farms that are certified by either ASC or BAP).

Of the aquatic species assessed in the Fair-Fish database, however, Nile tilapia is one of the few species that have been assigned the label “potential to be reared in good welfare” and were given the highest rating of 8 out of 10 (11). This is largely due to the abundance of literature on farming this species. Yet, while there is a large body of literature on the production of tilapia, including their feeding and spawning behavior, there is relatively minimal knowledge on welfare traits and indicators. It is likely the absence of knowledge that has prevented eco-labels and other certification bodies from being able to develop standalone animal welfare standards for farmed Nile tilapia.

Like all finfish, Nile tilapia are sentient beings capable of feeling pain (3). As such, it is crucial to ensure that they are provided with appropriate rearing conditions, and that any suffering caused by handling and slaughter procedures is minimised. This review aims to synthesise scientific knowledge on Nile tilapia, focusing on welfare considerations in common production systems. Topics covered include typical housing systems, environmental conditions, husbandry procedures, handling and transport, and slaughter practices, along with the associated welfare concerns and opportunities for this sector. We highlight key dimensions of Nile tilapia biology, including their ecological origins, social and reproductive behaviors, and physiological adaptations, to demonstrate how these factors should inform species-specific welfare guidelines.

### 1.1 The biology of the Nile tilapia

The Nile tilapia is a freshwater fish belonging to the Cichlidae family. The species originates in the Nile River and its associated water bodies in Northern and Eastern Africa. The species is highly adaptable and can thrive in diverse natural freshwater habitats, including rivers, lakes, irrigation channels, and even brackish water (4). Nile tilapia exhibit diurnal behaviour and are opportunistic omnivores, with a dietary spectrum encompassing phytoplankton, benthic algae, insect larvae, and even small fish (12, 13). In the wild, they can grow up to 29–60 cm in length, though this range is strongly influenced by ecological factors such as water temperature, habitat type, and food availability (14).

Farmed Nile tilapia are typically slaughtered at around 400–500 g, which they reach in 5–8 months, although they can live longer than 10 years and weigh over 5 kg (15). Nile tilapia typically reach sexual maturity in ponds at around 5–6 months, at which point, the males establish a territory by digging out a spawning nest (15). Females respond by spawning in the nest of a selected male, and the eggs are then immediately fertilised by the male, before the female collects the eggs into her mouth and moves away. The female incubates the eggs in her mouth and then broods the fry upon hatching for 1–2 weeks. Females feed little or not at all during this period. Once released, fry may swim back into the female's mouth when threatened (15).

Due to their preference for warmer waters, with a minimum temperature threshold of approximately 11–12°C, Nile tilapia have not successfully colonised temperate environments with colder water conditions (15). Nevertheless, they have undergone widespread distribution beyond their original introductions as a result of their use in aquaculture, proliferating in numerous freshwater and brackish tropical and subtropical ecosystems (4, 16). In fact, tilapia are generally considered among the most invasive and threatening species for aquatic ecosystems (4). Furthermore, this expansion has frequently resulted in hybridisation events with various other Oreochromis species, often leading to detrimental consequences for native aquatic populations (4, 17, 18). Despite the ecological impacts of these species, tilapia are widely farmed in countries with warmer waters, such as in Asia, and elsewhere in the world using artificially heated enclosures.

### 1.2 Nile tilapia production

Tilapia production is typically separated into two distinct phases: hatchery-based rearing of fingerlings and the subsequent grow-out of fingerlings to a marketable size, although in some systems, there may also be a third fattening phase for Nile tilapia (19). In the hatchery phase, brood fish are carefully selected for their production and genetic qualities, and controlled spawning is conducted to produce fertilised eggs (20, 21). The eggs are then hatched, giving rise to fry, which are reared to the fingerling stage.

The practise of rearing all-male cultures of Nile tilapia is common in the industry, as males grow faster than females, and keeping them in mono-sex groups prevents high energy losses through gonadal development and reproduction (22, 23). This is typically achieved by adding a derivative of the male sex hormone, 17α-methyltestosterone, to the fry's feed, which reverses the sex of female tilapia, resulting in an all-male culture (23, 24). However, there are increasing efforts to explore breeding procedures that do not rely on direct hormone use (15), such as high temperatures [e.g., (25, 26)].

At 2–3 months of age, or once the fingerlings have reached a size of approximately 30–40 g, they are moved to grow-out facilities where they remain until slaughter at around 5–8 months of age (15). Sexually mature males still perform territorial behaviours in the absence of females, including digging spawning nests and territory guarding (27, 28).

#### 1.2.1 Grow-out systems for Nile tilapia

The choice of production system used to farm Nile tilapia can substantially impact fish welfare and the environment surrounding the farm. For instance, housing systems may offer different volumes of space to the fish, impacting hierarchy development (29), and systems vary in how the generated waste impacts the surrounding water bodies (30).

Non-commercial extensive systems are typically based on small earthen ponds and are mainly used for subsistence (16). Commercial systems may also use earthen ponds, along with cages, tanks, raceways and recirculating systems, depending on the location and climate (4, 16).

##### 1.2.1.1 Pond systems

Earthen pond systems filled with freshwater are some of the oldest and most widespread systems used for Nile tilapia. However, to mitigate potential water pollution from feed wastage and fish metabolites, modern pond management increasingly employs sensor-based monitoring of dissolved oxygen, ammonia, and other parameters (31). Integrated multi-trophic aquaculture (IMTA) systems, where species such as shellfish or aquatic plants help remove excess nutrients, also show promise in reducing environmental impacts (32). Earthen ponds typically house monocultures, although polyculture ponds also occur (4). They range in size from 0.25–1 ha, with stocking densities up to 40,000 fish/ha (4 fish/m^2^) (33, 111, 121). Many earthen ponds produce fish in semi-intensive production systems with lower stocking densities that require fewer inputs (e.g., feed) (33). However, intensive monoculture production systems exist as well and can reach 30 tonnes/ha, although anywhere between 15–22.5 tonnes/ha is commonly seen in China, the largest producer of Nile tilapia (4). Escaped individuals from ponds are a significant concern and the main cause of invasive tilapia populations, hybridisation, and the spread of diseases to native wildlife (4, 7). The welfare implications of ponds depend on many factors, including water quality, control of pests, pathogens, and parasites, as well as management decisions regarding stocking density and feed. Although, some suggest that there is greater scope for maintaining fish health and achieving consistent performance in ponds compared with other systems (34, 35).

Water quality in earthen ponds is largely influenced by the external water source and feed used. While the mere presence of fish alone degrades water quality over time (112), necessitating water changes, the choice of water source and feed used can speed up water degradation and pose risks to fish. For example, some farmers with semi-intensive earthen ponds may use untreated water to supply the pond and may use manure and other potentially problematic feeds, leading to an increased risk of disease (113). Moreover, poor feeding practices that cause wastage may cause more rapid degradation of water quality (i.e., a decrease in dissolved oxygen and an increase in nitrites) (114). However, this suggests that, if water quality is consistently monitored, poor feeding practices in earthen ponds may be more quickly detected than in floating cages.

While earthen ponds provide substrate, which may act as environmental enrichment for tilapia, they are far shallower than the depth that tilapia might occupy in the wild. Pond depth can affect growth rate and mortality, with one study showing that a depth of 3 m led to a significant reduction in mortality (115). Deeper ponds may also provide access to a greater thermal gradient, allowing tilapia to seek deeper, cooler water on hot days or use behavioural fever to combat illnesses. The ability to access different depths of water may also allow tilapia to evade unpleasant stimuli (116). For example, Cerqueira et al. (116) demonstrated that more reactive tilapia sought lower temperatures (i.e., deeper waters), whereas proactive tilapia sought higher temperatures that were higher in the water column. This suggests that the depth of the average earthen pond may be detrimental to the welfare of tilapia.

Nile tilapia raised in pond systems are often grown to around 100–150 g with fertiliser alone (which produces algal blooms upon which the fish feed) and then given supplemented feed until they reach around 500 g (15). Semi-intensive farms may rely on high-quality feed, and some systems may adopt multiple grow-out phases to restock at lower densities, which, along with high-quality feed, high water exchange rates, and continuous aeration, produces larger fish destined for higher-end markets (15).

##### 1.2.1.2 Floating cages

Cage aquaculture represents a significant proportion of commercial aquaculture for fish, especially tilapia (36, 37). There are numerous types of floating net cages, and they are typically more intensive as they can subject the fish to high stocking densities (19, 38). Typically, cultures of Nile tilapia are kept at high densities in floating cages in reservoirs or lakes (15). However, one assessment of tilapia raised in earthen ponds and net cages found no significant differences between the welfare scores measured in the two culturing systems despite the cage systems being more intensive with higher stocking densities (37).

Cages prevent Nile tilapia from breeding, as males cannot carry out their natural nest-digging behaviors, and any eggs that females produce fall through the cage bottom, so mixed-sex populations can be reared in these systems (15). Compared with other techniques, these systems are popular as they allow for easy harvesting, close observation of the fish, and a relatively low capital investment (15). However, net cages leave the fish susceptible to predators, weather conditions, and a higher risk of disease outbreaks. Moreover, the fish are less tolerant of poor water quality due to stress caused by the higher stocking densities (15, 30, 38, 39).

##### 1.2.1.3 Recirculation systems

Specialised recirculation aquaculture systems (RAS) which are nearly always housed indoors have been engineered to enable tilapia's year-round farming in carefully controlled environments (15). These systems are designed to create optimal conditions for tilapia farming, regardless of the external climate, by efficiently recycling and maintaining water quality. RAS have water replacement rates ranging between 5%−15% a day, but they are energy intensive and generate a considerable volume of wastewater, leading to high capital and operating costs. Therefore, they are limited in their use both across species and contexts (15, 35, 40). However, these closed, super-intensive systems can have improved carbon and environmental footprints— especially when combined with renewable energy sources—and can reduce the impacts of nutrients, solids, and plastics on freshwater ecosystems (35, 40). Moreover, closed systems pose less of a biosecurity risk, as genetic contamination and the spread of pathogens to wild populations are reduced (35). The frequency of water exchange also means that water quality is easier to maintain because dissolved oxygen is frequently replenished and soluble organic matter and nitrates do not build up (15).

## 2 Welfare and production implications involved with commercial farming of Nile tilapia

### 2.1 Water quality and parameters

While Nile tilapia can withstand a wide range of environmental conditions, the optimal range for good welfare is far narrower. Optimal water quality parameters include water temperature of 25–32°C, dissolved oxygen generally above 5 mg/L, pH near 7–8, and total ammonia-nitrogen levels below 2 mg/L (15, 41). To maintain these optimal conditions, modern recirculating aquaculture systems (RAS) often incorporate advanced filtration (mechanical and biological), UV sterilisation, and real-time digital monitoring to regulate parameters. These technologies not only improve fish welfare but also reduce effluent discharge to the environment. Key factors such as temperature, flow rate, and salinity influence water quality and, in turn, affect the health and welfare of Nile tilapia. The welfare implications of current farming techniques in relation to water quality are discussed in the following sub-sections.

#### 2.1.1 Dissolved oxygen

Dissolved oxygen (DO) is one of the most critical environmental factors for fish, and like other water quality parameters, DO levels and consumption of DO, are influenced by several factors, including water temperature, stocking density, atmospheric pressure, and salinity (9). In Nile tilapia, inadequate DO (< 3 mg/L) can reduce feed intake, slow growth and heighten stress, thereby impacting immunity and overall health (42). Maintaining DO above 5 mg/L is recommended to optimise welfare. Moreover, as with other fish species, high temperatures increase DO consumption in Nile tilapia (9), as do handling procedures, which can cause DO consumption to double from 150% to 300% (43). Warm water cannot hold as much dissolved oxygen as colder water which exacerbates this phenomenon. High activity rates and stress causes an increase in metabolic rate as does the increase in temperature.

Fish naturally experience periods of hypoxia in their wild environments, particularly in stagnant water. Fish are also known to actively avoid water with low dissolved oxygen which can lead to physiological and psychological stress. Hypoxia can have numerous effects on fish, including retarding growth and feed utilisation, and thereby health (44). Furthermore, the negative production impacts of low levels of DO, such as stunted growth and feed intake, are further exacerbated by increasing stocking densities (42). Low DO levels also raise stress levels, which alter Nile tilapia's physiology and can have potential adverse knock-on effects on immunity, rendering it an important welfare concern (42). For example, when DO levels reach approximately 45% to 50% saturation (corresponding to 3.0–3.5 mg/L at a temperature range of 28–30°C), Nile tilapia employ a regulatory mechanism to diminish their metabolic activity (9). This response leads to a reduction in respiration and growth. Furthermore, DO saturations within the range of 10% to 20% (equivalent to 0.7–1.6 mg/L at temperatures spanning 26–35°C) result in significant discomfort and eventual mortality in tilapia (9). As a result, the chronic stress stemming from the metabolic slowdown caused by low DO levels can profoundly impact the growth and welfare of fish.

Although fish naturally have different coping strategies for dealing with periods of hypoxia, these coping mechanisms may negatively impact their health and susceptibility to disease (45). Therefore, Nile tilapia require optimum DO levels that are in accordance with their species requirements and the individual's size, as requirements change with size (44). Investigations suggest that the incipient DO level for Nile tilapia weighing between 60–100 g is around 3 mg L^−1^, and for individuals weighing between 200–270 g, is a higher level of 5.5 mg L^−1^ (46).

Dissolved oxygen is managed differently depending on the housing systems used for Nile tilapia. For instance, in tanks, raceways and ponds, aeration is typically used to improve DO levels, whereas as RAS systems are designed to replace 5%−15% of the water volume with new water each day, the high level of water exchange maintains satisfactory DO levels (15).

#### 2.1.2 Temperature

In the wild, adult Nile tilapia have a natural and preferred temperature range of 31–36°C, with lower and upper lethal temperatures of 11–12°C and 42°C respectively (15). Exposure to suboptimal temperatures (< 17°C or >36°C) can induce chronic stress and impair feed conversion, leading to reduced growth rates and compromised immune responses (15, 117). Nile tilapia only starts to spawn when the temperature reaches 24°C (15). Fry and early life stages typically require water between 25°C and 34°C, and optimal growth peaks at around 30°C (117). Juvenile fish require more restricted ranges of temperatures than adults, with temperatures below 17°C and above 35°C causing mortalities.

In farming systems, water temperature affects many other aspects, including dissolved oxygen, oxygen consumption, and the toxicity of ammonia (9). It can also directly impact metabolic rate, fin erosion, growth rates, morbidity and mortality of the fish themselves (15, 37). In welfare assessments of Nile tilapia, temperatures are often within the acceptable range for this species, primarily because of the flexibility and tolerance of the species to a wide range of temperatures (9, 21, 47). However, the increased toxicity of ammonia at higher temperatures suggests that other factors should be closely monitored depending on the temperature (9).

#### 2.1.3 pH

**Figure 1:**
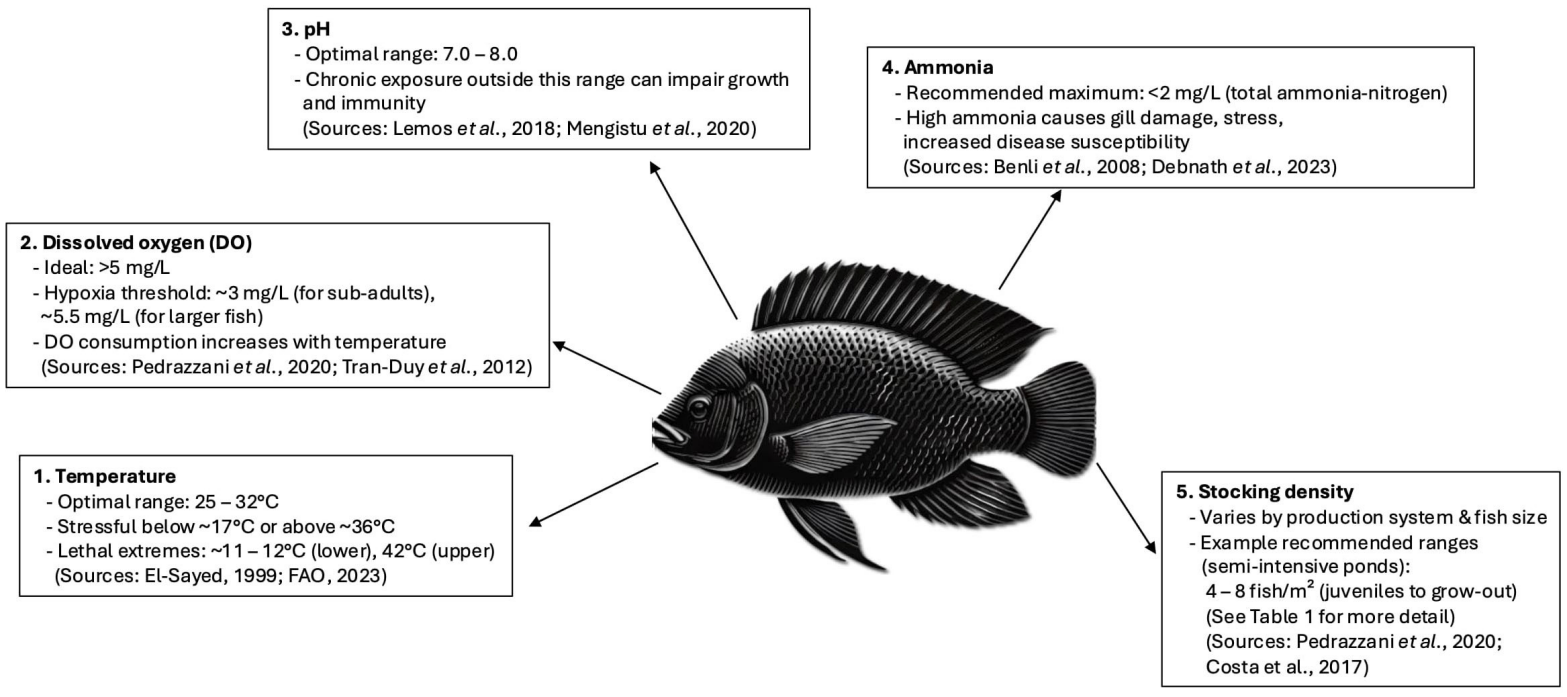
Illustration of the main physiological and environmental requirements for Nile tilapia (*Oreochromis niloticus*), highlighting optimal temperature, dissolved oxygen, pH, ammonia and stocking density ranges recommended for good welfare. Data adapted from El-Sayed (117), FAO (15), Pedrazzani et al. (9) and others as cited in the text.

The optimum pH level for Nile tilapia is thought to be between 7–8, with circumneutral (7.14) being optimum (Figure 1) (48, 49). Chronic exposure to highly acidic or alkaline conditions can damage gill structures, disrupt ion balance and increase susceptibility to pathogens (48). Although some suggest that Nile tilapia are tolerant to a wider range of pH (9), levels outside of this range can lead to tilapia suffering from a range of disorders, impeded development, morbidity and mortality (48). As with other aspects of water quality, pH is affected by other parameters, such as stocking density, which can increase the alkalinity of the water (48). pH also influences the nitrogen cycle as does the hardness of the water.

#### 2.1.4 The nitrogen cycle (nitrite, nitrate and ammonia)

The toxicity of ammonia is directly related to temperature; however, providing that temperatures are within the acceptable range, tilapia are relatively tolerant to ammonia (50). High ammonia (≥2 mg/L total ammonia nitrogen [TAN]), elevated nitrite or nitrate can impair oxygen uptake, cause tissue damage and elevate stress markers in Nile tilapia, reducing growth and overall welfare (41, 51). Ammonia is highly toxic to fish for many reasons but primarily due to the chemical burning of the gill lamellae which then reduces the diffusion of oxygen. Ammonia also interferes with ion channels, particularly potassium.

Despite this tolerance, controlling ammonia is still vital, especially in polyculture systems where some species commonly farmed with tilapia, such as the African catfish, can excrete more ammonia than tilapia (52). Unchecked, high ammonia levels can increase susceptibility to various bacterial, fungal, and parasitic diseases (7). Furthermore, one study found that exposure to sublethal ammonia concentrates of 2 mg l^−1^ total ammonia-nitrogen and above for 6 weeks caused histopathological changes in the gills, liver and kidneys (41). This led the authors to recommend that Nile tilapia be kept at levels lower than 2 mg l^−1^ to prevent tissue damage, which could make the fish more susceptible to disease (41).

Tilapia species are generally thought of as being more tolerant of nitrite than many cultured freshwater fish. Nevertheless, sublethal exposure to nitrite is known to have significant effects on haematological and biochemical parameters in Nile tilapia, causing increases in cortisol, glucose and cholinesterase (51). Like many other aspects of water quality, nitrite is affected by other parameters, including dissolved oxygen, stocking density, pH, and feeding rate (49). One study found that juvenile Nile tilapia are relatively tolerant to high nitrite levels in RAS systems, but that levels beyond 500 mg L^−1^ are likely to impede growth and negatively impact health (53).

#### 2.1.5 Salinity

Nile tilapia are relatively tolerant of certain levels of salinity and are considered euryhaline. However, they are not as tolerant as other species of tilapia, such as *O.aureus and O.mossambicus* (54). Brackish water farming (up to ~7 g/L) can be viable, provided fish are gradually acclimated. However, extreme salinity shifts can elevate cortisol levels and reduce feed efficiency (54). Gradual acclimatisation can be achieved by increasing salinity in increments of ~2 g/L^−1^ per day (54). Utilising sea or brackish water areas for fish farming can be economically important in regions where freshwater is scarce, and as a result, Nile tilapia's tolerance for salinity is often exploited (55). One study tested the tolerance of Nile tilapia to water salinities of up to 4,000 ppm and found that they can grow well under such conditions, especially when the pH is optimum (between 7–8) (55). Another study found that Nile tilapia could tolerate water salinity up to 7 g/L (7,000 ppm) without any negative impact on various haematological parameters or on the histological characteristics of their gills (54).

#### 2.1.6 Turbidity

Turbidity refers to the cloudiness of the water and is relevant to the fish's vision, particularly as fish housing systems can be naturally turbid due to high stocking levels (56). Moderate turbidity can actually enhance foraging success for tilapia by providing cover from predators, though extreme turbidity can hinder respiration and reduce visual cues for social interaction (56). Nile tilapia are well adapted to survive in waters with high turbidity (56). This is likely due to the fact they can switch to benthic feeding which suggests that in a RAS setup, there is no benefit to higher turbidity levels. Increasing turbidity by adding organic compost and clay materials can also reduce the toxicity of Chlorpyrifos, an organophosphorus insecticide which can enter water systems via agricultural run-off (57).

#### 2.1.7 Water exchange rate to maintain water quality

Another important consideration regarding the aquaculture environment for Nile tilapia is the rate of water renewal which is essential to maintain the good water parameters discussed in previous sections. While frequent water exchange improves oxygenation and waste removal, excessive water change may disrupt chemical signals used to maintain stable social hierarchies, thereby increasing aggression and lowering welfare. Balancing these factors is essential to minimise stress and optimise welfare (12, 58). For example, in pond systems where water is renewed or exchanged from the surrounding water bodies, the rate and extent of water renewal can affect the dominance hierarchy of Nile tilapia (29). This is because Nile tilapia use chemical cues in the form of hormones released into the water to mediate their social position (12). Likewise, one study found that, compared with a control tank, social instability among fish in tanks where the water was constantly renewed had greater social instability as the water washed away the chemical cues used for hierarchy maintenance (12). In particular, the subordinate fish kept in the tanks with continuously renewed water began to show significant increases in the number of attacks they made upon dominant individuals, compared with the individuals in the control tanks who showed no change in behavior (12).

Regulating flow rates in aquaculture systems is therefore critical for the welfare of tilapia, as too high flow rates can have psychological and physiological impacts on these fish. However, flow rates that are too low can also be damaging, and although tilapia are relatively hardy, prolonged substandard conditions can have negative welfare and production impacts (58). Typical flow rates of commercial RAS can vary between 1–3 tank volumes h^−1^, with some being higher (e.g., >5 tank volumes h^−1^) (58). Obirikorang et al. found that low water exchange (1.5 tank volume/h) systems were characterised by higher levels of ammonia, nitrogen and phosphate in the water and lower growth rates compared with the high exchange (6/h) systems (58).

There was also a higher prevalence of dermal ulcerations, oral lesions, and poor fin conditions in the low and medium tanks compared with the fish in the high exchange tanks (58). The fish in the low and medium tanks also tended to have higher haematological indicators of long-term oxygen stress and disease conditions than those in the high exchange tanks (58). As a result, the authors concluded that despite Nile tilapia typically being considered hardy and tolerant of a range of environmental conditions, their welfare is negatively impacted in conditions of low water exchange (58). As a result, they recommended that water replacement rates should be more than three times per hour. However, given the effect of continual water replacement on Nile tilapia social hierarchies and aggression (12), further research may be needed to establish the optimal water exchange rate for tilapia welfare.

### 2.2 Stocking density

The optimal stocking density for Nile tilapia depends on several variables such as life stage, water quality and rearing system, and inappropriate stocking densities (either too high or too low) can negatively impact welfare. The stocking and rearing densities for Nile tilapia in farming systems can significantly impact their welfare (Figure 1). Excessive crowding often leads to increased aggression, competition for resources, and higher stress hormone levels, which can impair immune function and growth (19, 59). Moreover, stocking density can impact water quality, which itself can impact the health and welfare of the fish (60). Recommended densities differ by system; for example, Pedrazzani et al. (9) suggest ~4–8 fish/m^2^ in semi-intensive ponds, and up to 20–30 kg/m^3^ in cages, though actual values must be tailored to water quality and fish size. Importantly, appropriate stocking densities are not fixed values but must vary according to the capacity of the production system to maintain good water quality. For example, systems such as recirculating aquaculture systems (RAS) and floating cages in open water can often support higher densities than earthen ponds due to their greater capacity for oxygenation, waste removal, and water exchange (123, 124). Overlooking this can result in poor water quality, leading to stress, disease, and welfare issues even at seemingly moderate densities.

Nile tilapia have a distinct social structure and a tendency for intraspecific aggression (60, 61), which makes stocking density in farming systems a particularly pertinent issue. In general, the link between higher stocking density and compromised welfare arises from issues such as increased competition for food, higher stress levels from crowding and greater chances of disease transmission (62). Given that the Nile tilapia is a social species, the number of interactions amongst conspecifics directly correlates with the number of individuals in a group. Generally, the larger the group, the higher the probability of fighting (60). In fact, one study showed that Nile tilapia fingerlings kept at high stocking densities (i.e., 35 or 45 fish in a tank measuring 30 × 40 × 100 cm) performed more aggressive behaviours and for longer durations, compared with fish in lower densities (59).

Conversely, the rate of aggression in Atlantic salmon is bell-shaped with lower aggression at lower densities, more aggression at medium densities and lower aggression at really high densities; this is because there is a behavioural shift in which it is no longer worthwhile for these fish to be aggressive at higher densities demonstrate reduced aggression at high stocking densities (63). Nile tilapia fingerlings also show more surfacing behaviour in high stocking densities than low and medium-density groups, as well as reduced activity and a lower final body weight (59). Other studies have also found that tilapia survival decreases as density increases, partly due to cannibalism but also due to the heightened risk of stress and susceptibility to disease that is associated with high stocking densities (7, 19, 64). However, one study found that even though higher densities were associated with lower weights, weight gain, and survivability, there was no impact on the physiological parameters of stress (19). Although, due to the complexities of comparing stocking densities between different commercial and experimental paradigms, it is difficult to draw conclusions (62, 65).

Table 1 presents a concise overview of stocking density recommendations extracted from the tilapia welfare assessment protocol, originally devised by Pedrazzani et al. (9). The protocol, originally adapted from the established Welfare Quality^®^ assessment methodologies employed in Atlantic Salmon farming, also served as the fundamental framework for the “Tilapia Toolkit” developed by WelfareMax (118). The purpose of this toolkit is to evaluate the potential risk of suboptimal animal welfare in tilapia farming ventures, particularly for investors seeking involvement in such operations (118).

**Table 1:** Production parameters for Nile tilapia farmed in ponds and cages.

**Raising system**	**Weight (g)**	**Age (days)**	**Stocking density (fish/m^2^) – no aeration**	**Stocking density (fish/m^2^) – with aeration or water renewal system**	**Food conversion ratio**	**Crude protein (%)**
Excavated pond	1–30	40–80	20–30	40–50	0.8–1.0	36–40
	30–300	80–120	4–5	6–10	1.2–1.3	28–32
	200–1,000	>120	0.8−1.2	2–3	1.4–1.6	28–32
Cage	1–30	40–90	1,200–1,500	1,200–1,500	0.8–1.0	40
	30–200	90–120	450–500	450–500	1.2–1.4	32
	200–1,000	>120	100–150	100–150	1.6–2.0	32

Adapted from Pedrazzani et al. (9) which is based on the RSPCA Welfare Standards for Farmed Rainbow Trout (109).

The recommended stocking density does vary based on the specific rearing system and the age and size of the tilapia being raised.

### 2.3 Environmental enrichment

According to the National Research Council's *Guide for Care and Use of Laboratory Animals*, environmental enrichment improves animals' welfare by providing them with “*sensory and motor stimulation through structures and resources that facilitate the expression of species-typical behaviors*.” It should be clarified that there are various types of enrichment such as structural, social, nutritional, sensory and cognitive. This section mostly focuses on the physical modifications to a tilapia's environment that are designed to provide complexity. Enrichment strategies (e.g., substrates, shelters) can reduce chronic stress, promote natural behaviours like nesting, and improve social cohesion among conspecifics. In practise, environmental enrichment for Nile tilapia can stimulate nest-building, reduce fear responses and enhance welfare indicators such as lower cortisol levels and improved feed conversion (66). However, careful design is crucial to avoid unintended increases in territorial aggression. Although research on environmental enrichment in Nile tilapia is still in its infancy, the value of environmental enrichment and its ability to stimulate natural, positive behaviors, is increasingly recognised in land-based farming. For example, providing substrate to chickens for dustbathing and foraging reduces fearfulness and intra-specific aggression and supports healthy skin and feathers (120) which has led to the provision of substrates in enriched cages and the increased popularity of cage-free aviaries. Despite the substantial positive impact that adequate environmental enrichment can have on fish welfare, it is often lacking in aquaculture systems, as housing design is typically informed by economic and ergonomic factors rather than the welfare needs of the fish (67).

However, the absence of environmental enrichment can be detrimental to the welfare in Nile tilapia. For instance, Nile tilapia raised in barren environments show increased behavioural indicators of fear in response to a novel object test, compared with fish raised in enriched environments with substrate and shelters (66). It should be noted that this differs between rearing systems. Enrichment is likely to be less valuable in natural outdoor ponds. Therefore, chronic stimulus reduction can result in these fish being hypersensitive to new stimuli, which can have considerable negative impacts for tilapia when they are exposed to new environments and stimuli as they pass through the different production phases. In particular, individuals raised in barren environments may suffer greater levels of fear and stress whenever they encounter a new experience, such as transport or handling, than would have been the case had they been reared in a stimulating environment. As Saraiva et al. (68) point out, the effects of domestication on farmed fish, including Nile tilapia, are still “weak,” and despite efforts and a limited degree of phenotypic change, their general needs and overall behavioural responses are still the same as their wild counterparts, and this can have implications for their welfare when kept in artificial environments. Recent studies also highlight the potential of computer-vision systems to quantify behavioural responses in enriched vs. barren environments, paving the way for data-driven refinements in enrichment protocols (69).

#### 2.3.1 Managing the territorial implications of providing resources to Nile tilapia

As Nile tilapia, like other cichlids, can be aggressive and territorial with conspecifics, this can impact how enrichment should be provided, especially as their social hierarchies tend to be closely interlinked with the structure of their environment. For instance, one study found that providing a full substrate coverage led to an increase in chasing behaviour in Nile tilapia, compared with partial or no substrate (68). However, they also found that bites were more prevalent when no substrate was provided. They also found that cortisol levels were the highest under the partial substrate condition and lowest in the full substrate condition (68). Their findings highlight the complexity and importance of providing species-specific environmental enrichment in aquaculture. They demonstrate that even though it may be challenging, there are still important welfare implications from either failing to provide suitable enrichment or not considering the species' needs.

Similarly, Barreto et al. (70) reported higher aggression in Nile tilapia males placed in novel environments with pebbles and artificial kelp compared with individuals placed in barren environments. The additional resources created a higher resource value, leading to increased aggression and decreased welfare (70). However, the opposite was found with redbreast tilapia, highlighting the importance of investigating species-specific differences (71).

Another study on Nile tilapia found that the provision of artificial water hyacinths only led to a non-significant increase in confrontations, compared with the control group, and that the provision of shelters led to a non-significant decrease in confrontations, compared with the control group (72). They also found that the provision of tryptophan led to a significant decrease in confrontations compared with all other treatments. Tryptophan is a food supplement that is known to improve social relationships in group-housed animals (73), reduce cortisol and aggression levels in fish (74), (75–77). The study from Neto and Giaquinto serves as an important reminder to examine the implications and benefits of providing enrichment from different perspectives, particularly the fishes' as the fishes demonstrated a clear preference for enriched environments that featured aspects of their natural environment (72). Furthermore, as Neto and Giaquinto pointed out, confrontation and a degree of socially-derived stress are natural experiences for wild Nile tilapia (72), and removing opportunities to execute these behaviours may cause even greater welfare concerns for these territorial fish. Nevertheless, as the captive environment differs from the wild environment, managing the provision of suitable enrichment is vital to ensure that it is species-specific and does not cause adverse responses.

#### 2.3.2 Stimulating natural behaviours

In intensive commercial systems like floating cages and RAS, substrate is often minimal or omitted entirely. As discussed, studies have demonstrated the importance of substrates for tilapia welfare, and its absence can negatively impact their welfare, rendering them more fearful of novel experiences (66, 78, 79). A lack of substrate can also disrupt Nile tilapia's natural reproductive cycles and their natural territorial behaviour. Male cichlids, including Nile tilapia, create spawning pits—or nests—in soft, muddy substrates (80). They will then defend these pits, and the wider mating territory (79). Substrate is, therefore, an important feature to facilitate natural behaviours in male Nile tilapia. Furthermore, male tilapia exhibit a preference for areas with substrate even in the absence of females and will still dig and defend nests, although such behaviour becomes more pronounced when females are present (27, 28, 78). Consequently, the presence or absence of substrate as a form of environmental enrichment holds significant implications when deciding between farming Nile tilapia in floating cages or RAS, where substrate is lacking, as opposed to earthen ponds, where substrate is abundant.

Preference tests have also demonstrated that as a benthic species, Nile tilapia can discriminate between the type of substrate and the colour of their surrounding environment (80, 81). For example, male Nile tilapia prefer to dig nests in lighter and more homogenous substrates (pure sand), over substrates composed of a mix of shells and sand, or stones with no substratum (80).

#### 2.3.3 Mitigating the negative implications of aquaculture

Husbandry practices in aquaculture, such as handling, cleaning, movement between housing systems, transport and slaughter, can all lead to stress in farmed fish, but there are efforts within research that seek to mitigate some of these negative implications. For instance, Nile tilapia are often farmed intensively, in high stocking densities that can result in stress and poor health. Bolognesi et al. (82) investigated the potential of tactile stimulation (scratches and touches by conspecifics or objects placed in the rearing environment) in reducing aggression between Nile tilapia. They found that stimulation reduced aggression in the fish but did not significantly affect their stress levels (82). Similarly, another study found that adding a brush that provided tactile body stimulation over a long period also failed to reduce cortisol levels in adult male Nile Tilapia, but did have production benefits (83).

Adding organic carbon-rich substrates to aquaculture systems can also mitigate some of the negative aspects of high stocking densities, and the addition of broken rice flour has been shown to promote anti-stress markers in Nile tilapia cultures (84). However, these steps cannot mitigate the impact of high stocking densities and limited space on the performance of natural behaviours and movement, and further research is needed to explore the impacts of behavioural restriction on the psychological wellbeing of Nile tilapia.

Farmed fish are periodically placed into new environments throughout their production lifespan, causing increased stress, especially if they have been raised in a barren environment (66). Therefore, providing Nile tilapia with a more complex and enriched environment can help to mitigate the effects of long-term neophobia. Similarly, when Nile tilapia are introduced to a new environment, water flow may act as a hydrodynamic enrichment that reduces the stressful effect of novelty on ventilation response whilst having no effect on cortisol levels (85). This means that although Nile tilapia are typically sensitive to water flow, when they are undergoing stressful relocation, it appears to reduce their ventilation rate, which is another indicator of stress response in fish.

Lighting and housing colour can also be used as environmental enrichment for Nile tilapia to mitigate the stressors of the captive environment. Studies suggest that Nile tilapia are affected by different light intensities, the colour of ambient light, and the colour of their housing (81, 86). For example, Maia et al. (81) found that juvenile Nile tilapia avoid red shelters but do not display a preference for any one colour. Similarly, Volpato and Barreto (86) found that blue light reduces the stress levels in confined Nile tilapia, compared with other colours (i.e., green or white), and that this effect was independent of light intensity. Carvalho et al. (87) also reported a positive relationship between light intensity and agonistic behaviours in Nile tilapia, and found that the cumulative effect of increased light intensity led to an increase in aggressive interactions. However, the effect was not significant enough to change the dominance ranking of the individual fish (87). Similarly, Tatemoto and Serra (122) also found that juvenile Nile tilapia were less aggressive when kept in low light conditions and concluded that artificially high luminosity could compromise their welfare.

As research shows, there are numerous ways in which environmental enrichment can be used to improve the welfare of Nile tilapia in commercial aquaculture. However, despite the growing body of research, Nile tilapia and many other commercially farmed fish are kept in barren conditions that fail to meet their welfare needs (67).

### 2.4 Feeding

#### 2.4.1 Feeding behaviour and diets of wild Nile tilapia

In the wild, Nile tilapia are opportunistic feeders and vary their diets depending on the season and their location (88–90). Gut dissections of wild Nile tilapia have revealed that they consume a mixture of organic detritus, zooplankton, and phytoplankton (89). They will also consume arthropods, with consumption increasing significantly during the wet season when they are more accessible (89). Nile tilapia can adapt to the abundance or lack of given food sources and can either passively philtre feed on plankton or actively pursue invertebrates (88–90).

#### 2.4.2 Diets of farmed Nile tilapia

While Nile tilapia can be extensively farmed without the need for manually adding formulated feed, as seen in integrated systems like rice field aquaculture. However, the predominant global approach to tilapia farming involves semi-intensive or intensive systems that depend on supplementary fertilisation and, in some cases, formulated feed (15, 91). An estimated 92% of tilapia farmed globally are given some combination of commercially formulated pelleted feed and/or feed produced on-farm to supplement their diets (10, 92). Pond fertilisation (that leads to plankton production upon which fish feed), along with these supplementary feeds, is also a common approach for producing low-cost, smaller tilapia (10, 15).

The impact of dietary protein levels on Nile tilapia growth and related factors has been extensively researched and depends on fish size, feed amino acid profile, and digestibility (91). Tilapia likely prioritise essential amino acids over total protein levels to meet their nutritional needs (95). For tilapia fry, the ideal crude protein ratio per kg of feed is approximately 450 g, decreasing to 350 g for fingerlings, and 250 g for adults (93, 94). High-protein diets with imbalanced amino acid profiles can lead to increased amino acid breakdown and greater nitrogen loss in the rearing environment (96). Therefore, given Nile tilapia's need for essential amino acids as opposed to dietary protein itself, replacement calculations should be carried out thoroughly in intensive systems that use artificial diets (91).

Furthermore, as wild tilapia consume a varied and changeable diet in the wild, there are welfare considerations when replacing these diets with manufactured and homogenous diets that only serve to meet the fish's nutritional needs, but not necessarily their behavioural needs or preferences. For instance, Nile tilapia prefer eating blue-green algae (89), and it is unclear how restricting Nile tilapia's feeding choices affects their mental wellbeing. The consumption of favoured foods is known to have hedonic effects in a range of species (97, 98). This may be true for Nile tilapia as well; however, more research is required to establish how the absence of favoured food choices affects Nile tilapia welfare.

There are different factors to consider in regard to the feeding practices of farmed Nile tilapia and their implications for welfare, such as the amount of feed, the timing, and the method of delivery. Optimised feeding strategies—such as multiple feeding points, demand feeders or automated feeding—help ensure all fish access feed, reducing aggression and improving uniform growth (91, 99). Much of the scientific literature is focused on production parameters rather than the welfare of the fish. Although welfare and production can be interlinked, the key drivers for much of aquaculture are associated with economics, and as feed represents the largest component of cost in tilapia production, this is a powerful factor in terms of decision making (49).

Underfeeding is a significant welfare concern, as not only is the motivation to feed and to satisfy hunger being thwarted but there are also physiological impacts (9). Unevenness in fish weight is also a production concern, and is often a cause of rejection (9). Underfeeding can be the result of the housing system and chosen feeding practices. For instance, in pond systems, if the feed is always given on one side, the more dominant individuals will prevent the subordinate ones from accessing it, resulting in a significant difference in feed consumption and subsequently vitality and health (99). Similarly, in cages, individuals near the surface tend to benefit from greater access to feed (99). Not only can underfeeding result in malnutrition, but it can also influence the behaviour of Nile tilapia, as agonistic behaviour and intense competition are likely to increase, leading to injuries (100). Different strategies can be employed to tackle these issues, such as using demand feeders that are spread out over the area, and at different depths, which can prevent dominant individuals from blocking others (33, 99). Furthermore, given that these fish are naturally hierarchical, taking time to study the distribution of fish in the culture, and their dominance behaviors, can provide helpful insight into how best to ensure all individuals can feed (9).

#### 2.4.3 Feed withdrawal and fasting

Like other fish species, Nile tilapia are typically subjected to feed withdrawal and fasting before transportation and slaughter (101). Short fasting (1–3 days) may be acceptable for gut emptying; however prolonged feed deprivation can heighten aggression, increase cortisol, and compromise welfare; further research is needed on the psychological impacts of fasting durations (101). Given that most existing studies are concerned with the physiological and production impacts. The duration of feed withdrawal varies with temperature and this is typically reported in degree days. Furthermore, intensively reared fish are often starved for far longer than required for various reasons, including beliefs around improving product quality (101). Further research is urgently needed into the psychological impacts of feed withdrawal and the optimum strategies for this practise, for example, whether immediate or gradual withdrawal is considered more humane.

### 2.5 Handling, transportation, crowding and slaughter

Farmed Nile tilapia are subject to multiple handling experiences throughout their lives, including during grading, transportation, and slaughter. During capture and slaughter, overcrowding, asphyxiation and live chilling without effective stunning are major welfare concerns, potentially causing prolonged distress (102). Handling any fish can be considered an extreme procedure, inducing acute stress due to their lack of familiarity with the experience. The procedure often results in injuries, especially since it typically involves removing fish from the water, further increasing the potential for harm (103, 104). Many of the welfare implications affecting Nile tilapia during these processes are universal to all farmed fish species and have been well covered elsewhere [e.g., (101, 104–106)], and so are not elaborated on here. It is worth mentioning here that tilapia are generally thought of as a hardy species.

There are, however, advances being made to mitigate some of the welfare concerns associated with handling and transportation, although their efficiency in doing so is often unclear. Recent advances, such as improved sedation techniques (e.g., clove oil, Aqui-S) and better-designed transport tanks with aeration and temperature control, can mitigate handling stress, though standardised protocols remain limited (103). Clove oil has been trialled as an anaesthetic for Nile tilapia during handling and transportation, as it can induce surgical anesthesia, characterised by a total loss of movement and minimum opercular movements, with a relatively quick recovery time and reduced cortisol levels (103, 107). It is efficient for up to 10 min in adult tilapia, which is helpful for routine handling procedures, although high mortality rates render it inappropriate for use when transporting juveniles (103). Ethical considerations regarding the use of anaesthetics in tilapia revolve around residue concerns, food safety, and the possibility that anaesthesia might be used to mask poor handling practices rather than improve them. Regulatory guidance often restricts certain anaesthetics in food fish (108). There are concerns about toxicity to both the animals and the humans that consume them.

#### 2.5.1 Slaughter

Currently, the vast majority of Nile tilapia are killed without humane methods, as proper or effective stunning procedures are not widely implemented. Stunning is defined as “any intentionally induced process that causes loss of consciousness and sensibility without pain, including processes resulting in instantaneous death” (119).

A recent review revealed that, although all interviewed Brazilian slaughterhouses and fish farms reported using pre-slaughter stunning for Nile tilapia, live chilling was cited as the most commonly employed method, used in 82% of the facilities surveyed. However, live chilling does not qualify as a stunning procedure, as it fails to induce an immediate loss of consciousness (102).

## 3 Conclusions

Given their prominence in the sector, we have sought to highlight some of the species-specific and most relevant welfare issues affecting Nile tilapia in commercial farms globally. This review is by no means an exhaustive attempt, as some subjects, including the use of hormones for sex-reversal and the developing area of genetic manipulation for improved strains, are worthy of whole papers in themselves, whilst still being victim to a lack of clear welfare focused research.

Future research should prioritise:

(1) refining humane slaughter protocols, including effective stunning methods that minimise suffering;
(2) employing sensor-based real-time welfare assessments that integrate water quality metrics and behavioural data;
(3) exploring novel feeds and precision feeding to reduce waste and improve fish health;
(4) developing species-specific environmental enrichment strategies that respect Nile tilapia's territorial and social nature; and
(5) establishing evidence-based breeding protocols that account for genetic and physiological welfare traits.

These innovations have the potential not only to improve fish welfare but also to optimise production efficiency, resource use, and product quality.

Moving forward, the most effective solutions will be those that align welfare improvements with better production outcomes. For example, precision feeding can reduce stress while improving feed conversion ratios; enrichment can reduce aggression while promoting uniform growth; and sensor-based monitoring can prevent welfare issues before they impact survival or product quality. By framing welfare as a driver—not a trade-off—of aquaculture performance, researchers and industry actors can accelerate uptake of humane, sustainable practices.
